# Improving Josephson junction reproducibility for superconducting quantum circuits: junction area fluctuation

**DOI:** 10.1038/s41598-023-34051-9

**Published:** 2023-04-25

**Authors:** Anastasiya A. Pishchimova, Nikita S. Smirnov, Daria A. Ezenkova, Elizaveta A. Krivko, Evgeniy V. Zikiy, Dmitry O. Moskalev, Anton I. Ivanov, Nikita D. Korshakov, Ilya A. Rodionov

**Affiliations:** 1 FMN Laboratory, Bauman Moscow State Technical University, Moscow, 105005 Russia; 2 Dukhov Automatics Research Institute, VNIIA, Moscow, 127030 Russia

**Keywords:** Quantum information, Qubits

## Abstract

Josephson superconducting qubits and parametric amplifiers are prominent examples of superconducting quantum circuits that have shown rapid progress in recent years. As such devices become more complex, the requirements for reproducibility of their electrical properties across a chip are being tightened. Critical current of the Josephson junction Ic is the essential electrical parameter in a chip. So, its variation is to be minimized. According to the Ambegaokar–Baratoff formula, critical current is related to normal-state resistance, which can be measured at room temperature. In this study, we focused on the dominant source of non-uniformity for the Josephson junction critical current–junction area variation. We optimized Josephson junction fabrication process and demonstrated resistance variation of 9.8–4.4% and 4.8–2.3% across 22 × 22 mm^2^ and 5 × 10 mm^2^ chip areas, respectively. For a wide range of junction areas from 0.008 to 0.12 μm^2^, we ensure a small linewidth standard deviation of 4 nm measured over 4500 junctions with linear dimensions from 80 to 680 nm. We found that the dominate source of junction area variation limiting ${\mathrm{I}}_{\mathrm{c}}$ reproducibility is the imperfection of the evaporation system. The developed fabrication process was tested on superconducting highly coherent transmon qubits (T1 > 100 μs) and a nonlinear asymmetric inductive element parametric amplifier.

## Introduction

As the complexity of superconducting quantum circuits grows with an increase of number of qubits, the tolerances for its frequency allocations are tightened to avoid frequency crowding^1^ or to obtain the exact interaction frequencies depending on the circuit architecture. Recent quantum processors and simulators already contain dozens and even hundreds of transmon qubits^2–9^ making qubit frequency reproducibility one of the crucial challenges. Transmon transition frequency follows $\mathrm{\hslash }{\mathrm{f}}_{01}\approx \sqrt{8{\mathrm{E}}_{\mathrm{c}}{\mathrm{E}}_{\mathrm{j}}}-{\mathrm{E}}_{\mathrm{c}}$, where ${\mathrm{E}}_{\rm{c}}=\frac{{\mathrm{e}}^{2}}{2\mathrm{C}}$ is charging energy and ${\mathrm{E}}_{\rm{j}}= \frac{\mathrm{\hslash }{\mathrm{I}}_{\rm{c}}}{2\mathrm{e}}$ is Josephson energy^10^. Qubit charging energy is accurately controlled, since capacitance $\mathrm{C}$ depends on the dimensions of a planar electrode which can be reproducibly fabricated with modern microtechnology. Critical current ${\mathrm{I}}_{\rm{c}}$ at zero temperature is related to the normal-state resistance ${\mathrm{R}}_{\mathrm{n}}$ by the Ambegaokar–Baratoff formula ${\mathrm{I}}_{c}=\frac{\pi \Delta }{2{\mathrm{eR}}_{\mathrm{n}}}$, where $\Delta$ is the superconducting energy gap^11^. The resistance can be measured at room temperature. ${\mathrm{I}}_{\mathrm{c}}$ of the tunnel junction, in turn, is defined by linear dimensions, tunnel barrier thickness, its microscopic structure and the material of the superconductor used. Typical nanoscale linewidth of Josephson junctions is challenging to reproduce with low variation using Niemeyer-Dolan technology^12,13^. Any deviation of the linear dimensions of the tunnel barrier directly affects qubit frequency. Moreover, some qubit architectures, for example, fluxoniums or $0-\uppi$ qubits require frequency tunability^14,15^. In this case, parallel tunnel-junctions with significant asymmetry (50:1–70:1) are introduced in order to obtain the desired Hamiltonian parameters and to reduce the sensitivity to flux-noise^16,17^.

On the other hand, parametric amplifiers based on superconducting nonlinear asymmetric inductive elements (SNAIL)^18^ and travelling wave parametric amplifiers (TWPA)^19^ require long arrays of multiple high asymmetry nonlinear elements, which determine their amplification range and saturation power. The above-described high asymmetry features require reliable design and fabrication of Josephson junction arrays with a wide range of areas (spanning from 0.01 to 1 μm^2^) on a single chip with the precise control of the small junction area. One could increase the area of the junctions with increased oxide thickness for better critical current control, but it leads to additional noisy two-level defects^20^.

In previous works dealing with Al-AlO_x_-Al junctions suitable for superconducting qubit fabrication, 2.5–6.3% normal resistance variation was reported for 0.01–0.16 μm^2^ junction area on a 76-mm wafer^21^. In another study^22^, the variation of 1.8% and 3.5% for ${\mathrm{R}}_{\mathrm{n}}$ of 0.042 μm^2^ was demonstrated for the junctions across 10 × 10 mm^2^ chip and 49 cm^2^ area, respectively. Although impressive results were obtained, the authors did not control linear dimensions. The assessment of junction fabrication reproducibility was carried out solely by measuring the resistance of the SQUID without tight control of the smaller junction. So, the asymmetry value was not controlled. It is worth noting, that fabrication of josephson junctions for superconducting qubit is rarely described in detail.

In this work, we report on improving fabrication reproducibility for small Josephson junctions (down to 80 nm) and investigate the origins of junction area non-uniformity. First, we strived to reduce the variation of linear dimensions of small junctions by optimizing e-beam lithography and get the standard deviation of linewidth less than 4 nm for resist Dolan bridge features width from 80 to 680 nm. Secondly, we minimized junction resistance variation and line edge roughness (LER) by optimizing the evaporation angle. As the result, we obtained 9.8–4.4% and 4.8–2.3% resistance variation for 22 × 22 mm^2^ and 5 × 10 mm^2^ chips respectively in a wide range of junction areas from 0.008 μm^2^ up to 0.12 μm^2^ using the standard Niemeyer-Dolan technique. Furthermore, our research shows a strong correlation of 0.82 between the junction area and the resistance, especially for small junctions of about 100 nm. We also show, that reproducibility is limited by the imperfection of the evaporation system.

## Materials and methods

The Josephson junctions (JJ), described in this work, were fabricated using the Niemeyer–Dolan technique. This method does not require large evaporation angles, unlike Manhattan junctions^23,24^, which leads to worse line edge roughness. Another advantage is a better suitability for fabrication of large arrays of junction, e.g., for fluxonium or parametric amplifiers, where junctions have to be placed directly one after another. However, this method has a poor Dolan bridge stability and narrow process window and, therefore, precise control of the fabrication operations is required. The recently developed subtractive methods for junction fabrication have shown promising results^25–28^. However, these methods require additional process steps with an extremely careful control of interfaces, which makes fabrication significantly more complicated. Nonetheless, the recommendations given in this paper apply for all the three methods.

For this study, 1 × 1 inch^2^ high high-resistivity silicon substrates (> 10,000 $\Omega \cdot \mathrm{cm}$) were used. Next, a 100 nm thick Single-crystalline Continuous Ultra-Smooth Low-loss Low-cost (SCULL) ^29^ Al base layer is deposited with ultra high vacuum (UHV) e-beam evaporation system. Probe pads were defined using a direct-laser lithography and then dry-etched in BCl_3_/Cl_2_ inductively coupled plasma. Resist bilayer for junction itself is composed of MMA(8.5)MMA copolymer (500 nm) and chemically amplified resist CSAR 62 (100 nm).

**Figure 1:**
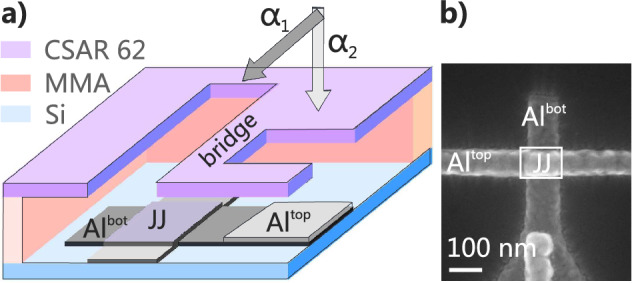
Niemeyer–Dolan bridge technique used in this paper. (**a**) Scheme of the used shadow evaporation process. The first metal layer was deposited at an angle (dark grey color) through two-layer resist mask MMA-CSAR, and the second layer at 0 angle (light grey color). Angular deposition was performed first, in order to avoid shading from the junction wall at the second deposition, causing electrode discontinuity. (**b**) Top view SEM image of the fabricated junction after the removal of the resist mask.

Resist thicknesses were precisely controlled to minimize junction area variation. Resist thickness range $3\upsigma$ is typically less than 5 nm. The Josephson junctions are then defined using a 50 kV electron-beam lithography tool. The development was performed manually in a bath of amilacetat at room temperature followed by a 60 s IPA dip and addittional 240 s in a IPA:DIW solution to get 200 nm undercut. We use oxygen descum followed by buffered oxide etchant (BOE) dip in order to remove e-beam resist residues and native oxide. Aluminum junction electrodes were e-beam shadow-evaporated in a UHV system. The first electrode was evaporated at ${\mathrm{\alpha }}_{1}$ angle, which we varied in our experiments. Then the electrode was statically oxidized at 5.2 mBar during 10 min. The second electrode was evaporated at ${\mathrm{\alpha }}_{2}=0$ angle, (see Fig. 1) for the cartoon of the process. Thicknesses of bottom and top electrodes are 25 nm and 45 nm, respectively. Resist lift-off was performed in a bath of *N*-methyl-2-pyrrolidone at 80 °C for 3 h and rinsed in a bath of IPA with sonication. Junction normal resistance was measured with an automatic probe station.

## Experimental overview

The dominant source of junction resistance non-uniformity is junction area variation^22^. The deviation of the mask feature size could be minimized by optimizing e-beam lithography (EBL) process^30,31^. There is typically a large junction wiring area (1–25 μm^2^) leading to significant backscattering exposure of Dolan bridge during EBL. We simulated proximity effect using Monte Carlo method for the two different mask stacks: MMA-PMMA A4 and MMA-CSAR 62. The simulations indicate that the dose on the junction area increases by ~ 30% due to backscattering exposure, that corresponds to widening of the feature size by 50 nm in case of PMMA A4 top layer. The backscattering for the highly sensitive AR-P 6200.04 was three times lower. In the experiments, we used a high-sensitivity resist to minimize backscattering and fixed wiring design to keep linewidth (LW) biases constant.

**Figure 2:**
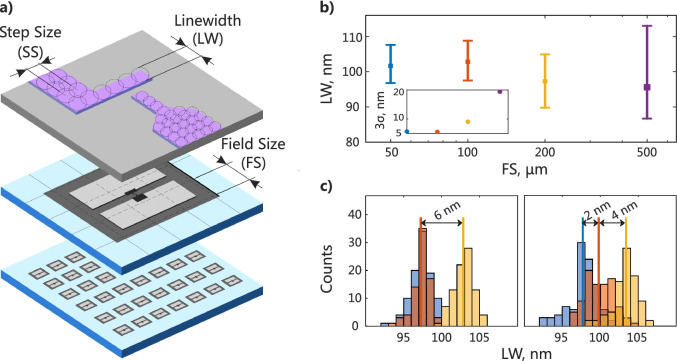
(**a**) Electron beam lithography process. From bottom to top: test chip, enlarged image of a single cell with a junction in the center, hidden image of the Josephson junction in the resist. (**b**) Measured linewidth (LW) variation of the nominal 100 nm feature size grouped by field size. Inlet shows 3σ of linewidth variation. Dot color corresponds to the bars in the main picture. (**c**) Histograms of measured linewidths for 100 nm (blue), 103 nm (red) and 105 nm (yellow) nominal dimensions. When lines are scanned longitudinally (left picture), close nominal linewidth are undistinguishable, since their LW histograms overlap completely and average values are the same. The lines scanned transversely (right) have LW average values difference corresponding to the beam step size.

First, we optimized e-beam lithography: writing field size (FS) and step size (SS) to study LW variations by measuring more than 1200 junctions. Direct scanning electron microscopy (SEM) measurement of the resist mask LW is challenging, since the electron beam damages the resist mask and distorts the results. To overcome this problem, we instead measured the width of sputtered at 0° “shadows” of the mask features using an automatic SEM tool. Our test chip contained 98 junctions for each of the the patterning parameter: field size (50, 100, 200, 500 μm), scanning direction (longitudinal and transverse), step size (2, 3, 5 nm) and linewidths: (100, 103, 105, 150, 300, 500 nm). The exposed area of the topology is divided into square write fields—maximum area that can be written at fixed stage position with the size FS. We minimized writing field size (FS) in order to lower beam deflection and therefore lower aberrations, which increase LW variations and edge roughness. A cartoon of the layout exposure process is shown on (Fig. 2a). With the smaller FS of 100 μm instead of 500 μm the linewidth 3σ variation decreases from 17.4 to 7.1 nm. For FS less than 100 μm the LW variation does not change (see Fig. 2b). We noticed the lowering of maximum positioning deviation from 41 to 33 nm for 500 μm and 200 μm field size, respectively, that may be explained by reduction of spherical aberrations.

Within the field size the pattern is scanned with a step size, which is limited by pattern generator frequency. The calculated step size equals to 2 nm for our 50 MHz tool with 180 μC/cm^2^ and 200 pA beam current. We tried to obtain the highest accuracy corresponding to our step size. We did not observe any differences between exposed nominal feature sizes of 100 nm and 103 nm, when e-beam scanned parallel to the longest edge of a feature (longitudinally). We used another scanning algorithm that scanned the junctions transversely and then the difference of 2 nm between feature sizes reproduced (Fig. 2c). The measurement results for the other nominal linewidths are listed in the supplementary materials.

**Figure 3:**
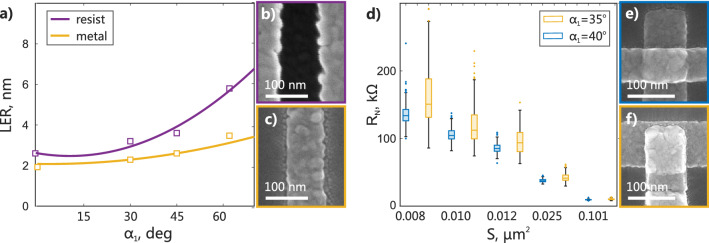
(**a**) Line edge roughness of resist mask after evaporation (purple) and bottom electrode (yellow) as a function of deposition angle (**b**) SEM image of resist mask after evaporation at ${\mathrm{\alpha }}_{1}=$ 62°. (**c**) SEM image of measured bottom electrode evaporated at ${\mathrm{\alpha }}_{1}=$ 62°. (**d**) Measured normal resistances grouped by nominal junction area. Blue color corresponds to junctions evaporated at 40° angle with electrodes fully overlayed. Yellow-junctions evaporated at 35° with incomplete electrodes overlay. (**e,f**) SEM images of 0.01 μm^2^ junctions used in the experiment with and without full overlay respectively.

Next, we experimentally investigated the influence of deposition angle on the mask LER. We automatically measured LER of the resist mask and the bottom electrode. We used Matlab and ImageJ for image recognition and LER quantification. Line edge roughness of the electrodes grows slowly from 2 nm for the deposition angles from 0° to 45° up to 4 nm, and dramatically increases starting from 45° (Fig. 3a). Such a big LER values can affect the ${\mathrm{I}}_{\mathrm{c}}$ variation of comparably small junctions (sub 100 nm), which are common for many devices.

After the first junction electrode deposition at an angle, there is a rough metal layer deposited on the resist mask walls (Fig. 3b), which increases LER of the resist mask. This increased resist mask roughness transfers into the first (Fig. 3c) and second deposited electrodes. However, smaller evaporation angle could lead to unstable overlay of the electrodes which results in additional resistance variation (Fig. 3d). We experimentally compared two different evaporation angles ${\mathrm{\alpha }}_{1}$ of 40° with full overlay (Fig. 3e) and 35° with partial overlay (Fig. 3f) by measuring 5000 Josephson junctions with areas from 0.008 to 0.12 μm^2^. There is the factor of two improvements in resistance spread for the electrodes with full overlay. Out theory is that there are slightly different evaporation angles across the substrate due to evaporation system imperfectness. This imperfection lies in the fact that the source of evaporation is a point, therefore the flow of the evaporated metal has a conical shape. Because of such shape of the metal flow, the deposition angles across the wafer are distorted^32^. The margin for junction electrodes overlap compensates this angle variation. The overlay margin can be increased by reducing the width of the bridge, but minimal bridge width is limited by its stability. We used 150 nm wide bridge in our experiments, as it provides robustness for the whole range of junction areas.

## Results and discussion

**Figure 4:**
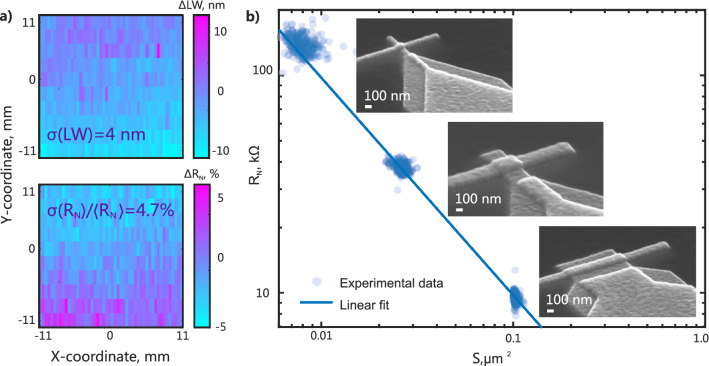
(**a**) Heat maps of measured top electrode LW and resistance RN for 150 × 170 nm^2^ junctions. For both the LW map and the resistance map, there is a visible gradient stemming from evaporation source imperfection. (**b**) Measured resistance vs. measured junction area. Junctions were fabricated in three area groups: 0.008, 0.025 and 0.12 μm^2^. Solid line is a linear fit. SEM images of the junction in the corresponding junction area groups. SEM-images show fabricated Josephson junctions with corresponding 0.008, 0.025 and 0.12 μm^2^ areas.

For a quantitative investigation of JJ reproducibility across the substrate, we fabricated 22 × 22 mm^2^ chip with a statistically significant number of Josephson junctions (> 5000) and measured their normal resistance at room temperature. The substrate contained six 5 × 10 mm^2^ chips with different junction areas: 0.008, 0.010, 0.012, 0.025 and 0.120 μm^2^. (Fig. 4a) shows heat maps of measured resistances and LWs across the sample, fabricated with the following parameters: 100 nm field size, 2 nm step size, 40° evaporation angle. Resistance spread across the substrate is 4.4–9.8% for JJ areas 0.120–0.008 μm^2^, the average inter-chip variation is 3.1–6.3%. Furthermore, the average variation is 2.3–4.8% across single 5 × 10 mm^2^ chip. Substrate-scale chip LW standard deviation σ for 150 × 170 nm^2^ junctions is about 4 nm. (The heat maps of the other junction areas can be found in supplementary materials) (Fig. 4b) shows normal resistance for the directly measured JJ areas.

The slope of the linear fit is −1, since $\mathrm{R }= 1/\mathrm{A}$, where A is the area of the junction. The correlation coefficient between the normal resistance and the JJ area is 0.82, thus the main contributor in ${\mathrm{R}}_{\mathrm{n}}$ fluctuation is LW variation. We speculate that the other source of resistance spread is oxidation inhomogeneity. The oxidation inhomogeneity depends both on the microstructure of the electrode surface^22,33–35^, which depends on the deposition parameters, and on the oxidation parameters of the tunnel barrier, such as the process time, oxygen pressure, and the oxidation method^32,36^. We observe a linearly dependent gradient of resistance on the heat map, which is also reproduced on LW heat map. We assume that the gradient of ${\mathrm{R}}_{\mathrm{n}}$ originates from the imperfection of the evaporation source. To test this hypothesis, we fabricated another substrate, with the both electrodes evaporated at 0° angle to eliminate any significant angle variations. LW variation gradient became less visible. The LW standard deviation σ at 0° angle was lower than at 45°: 3.3 nm against 4 nm (the heat map is presented in the supplementary materials). A possible practical solution to eliminate the gradient could be to use a bias of the resist mask linewidth features to compensate the junction area variation. To summarize, our Junctions resistance variation is mostly limited by junction area fluctuations, which, in turn, are limited by evaporation system imperfectness and resolution of our 50 keV e-beam lithography tool.

To test the developed process, we fabricated and measured two similar chips with 6 single transmon qubits each using the optimized lithography and evaporation parameters for junctions described in this work.

We also deposited bandages to shortcut the parasitic junctions^37^ and placed free-standing airbridges across microwave lines in order to supress the parasitic modes. Two qubits on the chip were frequency-fixed, the other had various SQUID asymmetries α (0.25, 0.35, 0.45, 0.55). The areas of 250 × 260 nm^2^ for single junction and 200 × 220 nm^2^ for the larger junction of the asymmetrical SQUIDs were used. The chips were wire-bonded in the Cu boxes and measured in dilution refrigerator at 20 mK. Coherence and frequency masurements are presented in Table 1. Next, we fabricated and tested a SNAIL parametric amplifier with 25 SNAIL cells and large asymmetry^38^. The areas of 0.63 μm^2^ and 0.055 μm^2^ for large and small junctions respectively were used (α = 0.087) to obtain the frequency of 6 GHz.

**Table 1:** Properties of the fabricated devices.

Device	Parameter	Average
Qubit chip No. 1	T_1_, μs	129.3 ± 19.5
	T_2_*, μs	45.7 ± 18.6
	f01, GHz	4.46 ± 0.12
	$\Delta {\mathrm{f}}_{01}$, %	2.06
	$\mathrm{\Delta \alpha }$, %	8.0
Qubit chip No. 2	T_1_, μs	190.4 ± 67.5
	T_2_*, μs	16.7 ± 10.0
	f01, GHz	4.32 ± 0.08
	$\Delta {\mathrm{f}}_{01}$, %	1.37
	$\mathrm{\Delta \alpha }$, %	10.5
Parametric amplifier	Δf, %	3.2
	Δα, %	3.3

## Summary and conclusions

To improve Josephson junction reproducibility, we performed a systematic study of fabrication process origins of critical current non-uniformity, focusing on junction area fluctuations. To that end, we fabricated a significant number of junctions and directly measured their dimensions and normal resistance. A high-sensitivity resist stack was picked to lower the backscattering exposure. We minimized the e-beam lithographer field size to improve junction linewidth variation and used a proper scanning algorithm to increase accuracy. Evaporation angle showed a direct effect on the junction line edge roughness. LER of the resist mask transfers to the deposited junction and grows with increasing evaporation angle. We provided a complete junction electrode overlay and improved resistance variation compared to the incomplete overlay. The developed fabrication process demonstrated 9.8–4.4% and 4.8–2.3% resistance variation for 22 × 22 mm^2^ and 5 × 10 mm^2^ chips respectively in a wide range of junction areas from 0.008 μm^2^ up to 0.12 μm^2^. We found a strong correlation of 0.82 between the normal resistivity and the junction area. Junction reproducibility is dominantly limited by the fluctuations of the resist mask linewidth and could be further improved by using a lithography tool with a higher resolution. We have also noticed that the evaporation system imperfectness distorts junction area and therefore increases ${\mathrm{I}}_{\mathrm{c}}$ variation.

## Supplementary Information

Supplementary Information.

## Data Availability

The datasets used and/or analysed during the current study available from the corresponding author on reasonable request.
