# Supplementary material for: Improving Josephson junction reproducibility for superconducting quantum circuits: junction area fluctuation
Source: Sci Rep. 2023 Apr 25;13:6772. doi: 10.1038/s41598-023-34051-9 (PMC10130087; doi:10.1038/s41598-023-34051-9)
Supplement: Supplementary file 1 — Supplementary Information. [file 41598_2023_34051_MOESM1_ESM.docx]

**Supplementary Information**

**Improving Josephson junction reproducibility for superconducting quantum circuits: junction area fluctuation**

*Anastasiya A. Pishchimova,1,2,a) Nikita S. Smirnov,1,a) Daria A. Ezenkova,1 Elizaveta A. Krivko,1 Evgeniy V. Zikiy,1 Dmitriy O. Moskalev,1 Anton I. Ivanov,1 Nikita D. Korshakov,1 and Iliya A. Rodionov1,2**

^1^FMN Laboratory, Bauman Moscow State Technical University, Moscow, Russia

^2^Dukhov Automatics Research Institute, (VNIIA), Moscow, Russia

**Resistance and linewidth measurements**

Test junctions were defined into resist bilayer of 500 nm MMA(8.5)MAA copolymer and 100 nm of CSAR 62 using followed exposure parameters: 100 um field size and 2 nm step size (see Materials and methods). Next, we deposited 50 nm of Al at 0° angle, performed lift-off and measured the “shadows” of the mask features using an automatic SEM tool. Our test chip contained 98 junctions of different nominal top electrode linewidths: 100, 103, 105, 150, 300, 500 nm. The measurement results for different scanning algorithms (across the electrode and along the electrode) are presened in Table 1.

| *Table 1. Measurements of resist mask linewidth* | | | | | | | | | | | | |
| --- | --- | --- | --- | --- | --- | --- | --- | --- | --- | --- | --- | --- |
| **Scanning direction** | **along** | | | | | | **across** | | | | | |
| **LW_nom,_ nm** | 100 | 103 | 105 | 150 | 300 | 500 | 100 | 103 | 105 | 150 | 300 | 500 |
| **LW, nm** | 99 | 99 | 104 | 150 | 302 | 502 | 96 | 99 | 101 | 144 | 300 | 500 |
| **3σ, nm** | 4.4 | 4.4 | 5.1 | 5.5 | 6.0 | 6.6 | 4.5 | 4.4 | 5.8 | 8.3 | 5.6 | 5.2 |
| **N** | 98 | 98 | 96 | 97 | 95 | 97 | 93 | 94 | 84 | 88 | 89 | 98 |

The heat maps of both top and bottom electrodes linewidths and junctions resistance across the 22x22 mm^2^ area are presented in Table 2. Mean measured value with 99.7% confidence limit is shown on the corresponding heat map. We obtained the gradient in resistance, which is also reproduced in top electrode linewidth. We assumed that the gradient originates from evaporation source imperfectness. To test this, we fabricated another substrate and evaporated both electrodes at 0° angle to eliminate any significant angle variations. The heat maps of features linewidths are shown in Table 3. The gradient in the top electrode linewidth was no longer visible and the variation was lower in comparison to the electrodes evaporated at an angle.

| *Table 2. Measurements of fabricated josephson junctions* | | |
| --- | --- | --- |
| **area** | **linewidth** | **resistance** |
| **0,008 um^2^** | 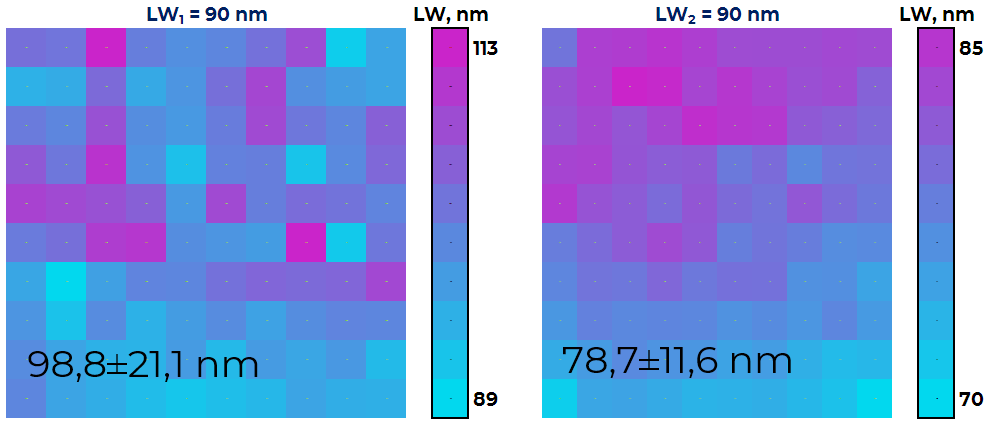 | 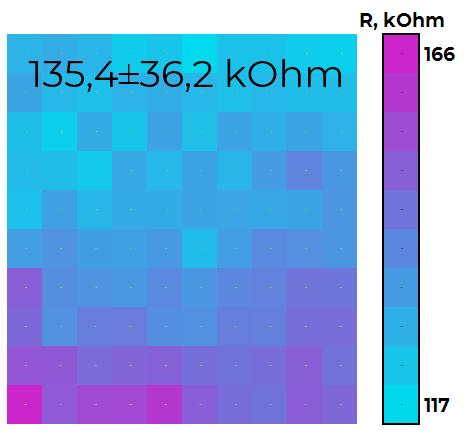 |
| **0,025 um^2^** | 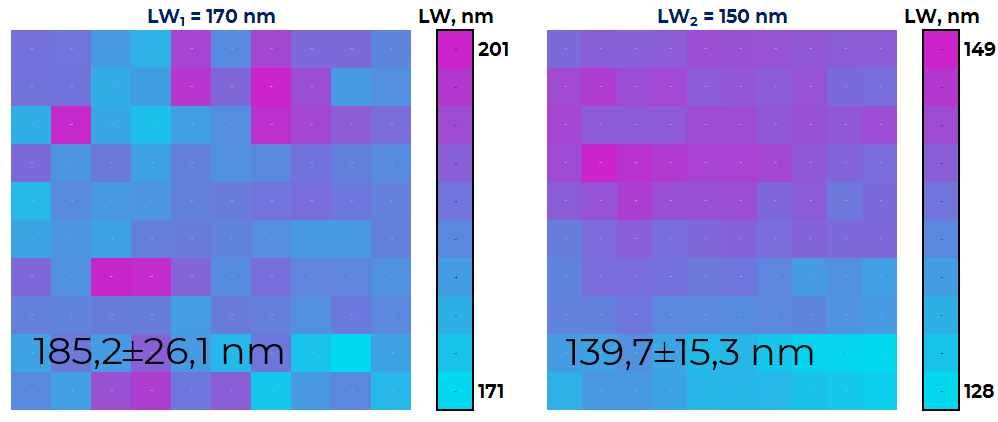 | 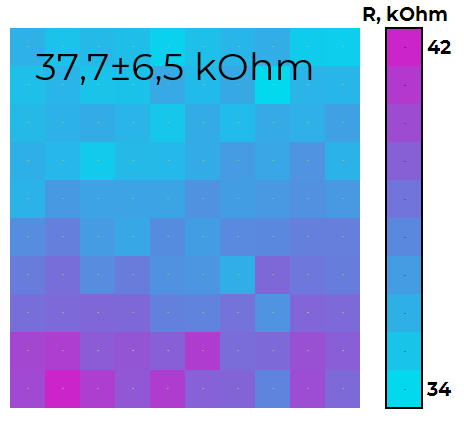 |
| **0,12 um^2^** | 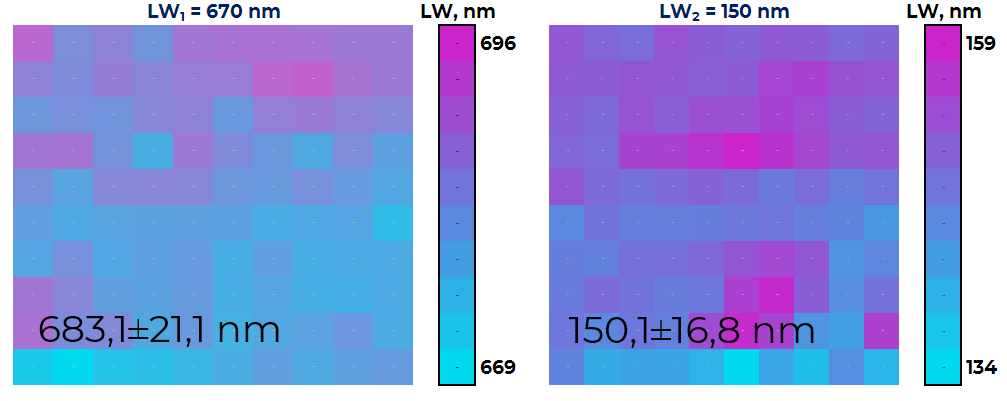 | 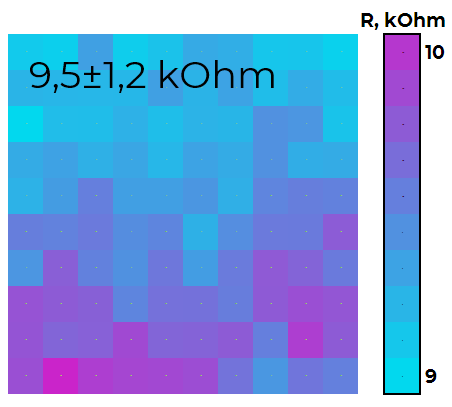 |

|  | *Table 3. Measurements of resist mask linewidth* |
| --- | --- |
| **area** | **Heat map** |
| **0,008 um^2^** | 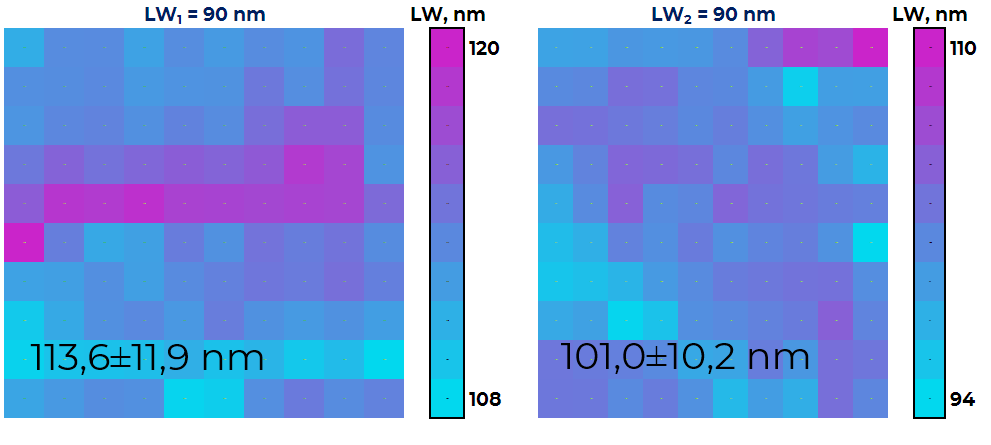 |
| **0,025 um^2^** | 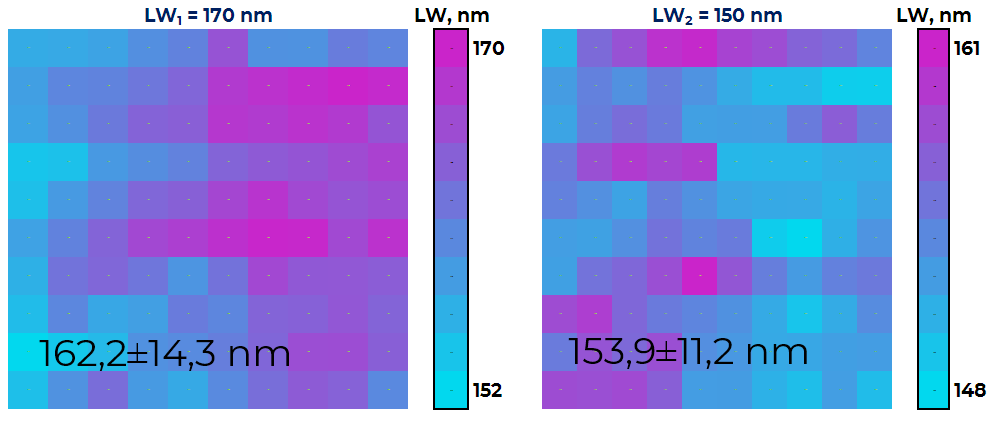 |
| **0,12 um^2^** | 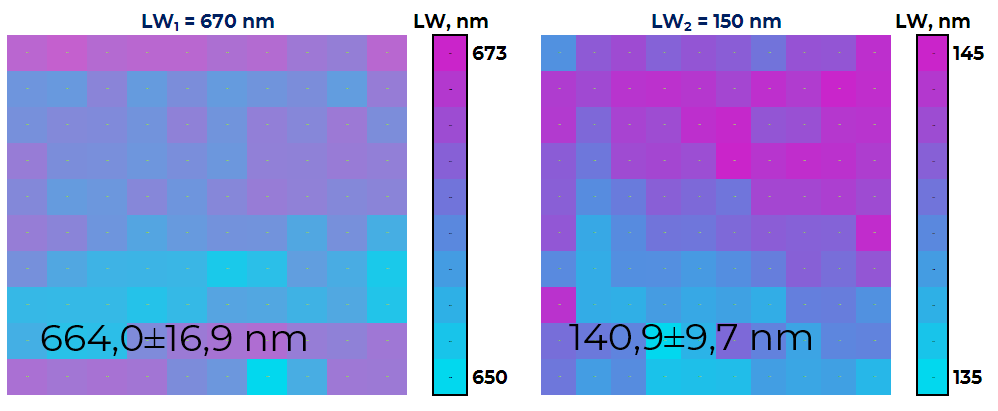 |
